# Supplementary material for: Feasibility and preliminary efficacy of heavy Lifting Strength Training versus Usual Care in Head and Neck Cancer Survivors (the LIFTING 2 Trial): A study protocol for a single-centre, phase II, randomized controlled trial
Source: PLoS One. 2025 Jul 10;20(7):e0326860. doi: 10.1371/journal.pone.0326860 (PMC12244484; doi:10.1371/journal.pone.0326860)
Supplement: Attachment — Submitted filename: Response to Reviewers.docx [file pone.0326860.s003.docx]

PONE-D-25-15890

Feasibility and Preliminary Efficacy of Heavy Lifting Strength Training versus Usual Care in Head and Neck Cancer Survivors (the LIFTING 2 Trial): a study protocol for a single-centre, phase II, randomized controlled trial

PLOS ONE

JOURNAL REQUIREMENTS

**1.  Please ensure that your manuscript meets PLOS ONE's style requirements, including those for file naming. The PLOS ONE style templates can be found at**

<https://journals.plos.org/plosone/s/file?id=wjVg/PLOSOne_formatting_sample_main_body.pdf>  and

<https://journals.plos.org/plosone/s/file?id=ba62/PLOSOne_formatting_sample_title_authors_affiliations.pdf>

The manuscript and file names have been revised according to PLOS ONE style requirements. Our apologies for the previous errors.

**2. Thank you for stating the following in the Funding Section of your manuscript:**

***“Dr. Kerry Courneya will be fully funding this trial out of his Canadian Institutes of Health Research Foundation Grant and his Canada Research Chair funding. This will cover costs of study personnel including casual employees and research assistantships for graduate students. It also includes incidentals such as printing letters and study brochures to mail out for recruitment, cleaning supplies, and transportation to and from our facility. Participants will be reimbursed for all study-related expenses. We have 3 pre-paid parking spots at the University of Alberta which are reserved for participants. If unavailable, they will be reimbursed for all parking costs. CMP was partially funded through Canada’s Research Chair program.”***

**We note that you have provided funding information that is not currently declared in your Funding Statement. However, funding information should not appear in the Acknowledgments section or other areas of your manuscript. We will only publish funding information present in the Funding Statement section of the online submission form.**

**Please remove any funding-related text from the manuscript and let us know how you would like to update your Funding Statement. Currently, your Funding Statement reads as follows:**

“The author(s) received no specific funding for this work.”

Our apologies for this inconsistency. All funding-related text has been removed from the manuscript. Please update our Funding Statement with the following:

*Dr. Kerry Courneya will be fully funding this trial out of his Canadian Institutes of Health Research Foundation Grant and his Canada Research Chair funding. This will cover costs of study personnel including casual employees and research assistantships for graduate students, and the cost to conduct bioelectrical impedance analyses for the body composition assessment of all study participants, at baseline and post intervention. Dr. Courneya’s funding will also cover the costs of incidentals such as printing letters and study brochures to mail out for participant recruitment, cleaning supplies for the laboratory, and transportation to and from our facility for all study participants. Dr. Carla Prado was partially funded through Canada’s Research Chair program.*

**Please include your amended statements within your cover letter; we will change the online submission form on your behalf.**

Our amended statement above has been added to the cover letter. We appreciate you changing the incorrect information within the online submission form.

REVIEWER #1:

**Thank you for the opportunity to review this manuscript titled "Feasibility and Preliminary Efficacy of Heavy Lifting Strength Training versus Usual Care in Head and Neck Cancer Survivors (the LIFTING 2 Trial): a study protocol for a single-centre, phase II, randomized controlled trial." This is a very strong protocol paper that clearly outlines the rationale, objectives, and methodology of the project. Feasibility studies such as this are extremely important to establishing strong evidence-bases and unfortunately are largely underutilized. I look forward to reading about the results of this study and how the intervention evolves throughout the research process. I have two main suggestions for improvement:**

**1. Your stated primary outcome is feasibility though you are powering on the efficacy outcome. This is common in implementation trials as well where we cannot power on the implementation outcome, therefore we power on the intervention outcome. It would be good to justify this choice.**

We have now justified our choice to power our study on muscular strength as an intervention outcome. As noted by the reviewer, it is difficult to power a study on the feasibility outcomes so we have powered the study on the first indicator of efficacy, which is muscular strength. We have indicated this on page 21 of the manuscript.

**2. I suggest adding plans for handling missing data in the analysis to the statistical considerations section.**

Thank you for this suggestion. On Page 22 of the manuscript, we explain that if missing data is <10% we will conduct a complete case analysis. If missing data is >10%, we will employ a multiple imputation missing data strategy. There will be no interim analyses (i.e., at 6 weeks/midway through the 3-month intervention).

**There are very minor grammatical/spelling errors in a few places. I would recommend a read-through of the entire manuscript and possibly use of grammar-checking software to help clean these minor errors.**

A thorough read through of the entire manuscript has been completed, as well as the use of a grammar-checking software.

REVIEWER #2:

**The LIFTING 2 Trial presents a relevant and timely study evaluating the feasibility and preliminary efficacy of heavy lifting strength training (HLST) in head and neck cancer survivors. The randomized controlled design, inclusion of objective and patient-reported outcomes, and structured intervention protocol are commendable. From a methodological and statistical point of view, I have the following comments:**

1. **The sample size calculation is based on detecting a large effect size (Cohen’s d = 0.80) with 80% power and α = 0.05, resulting in 38 evaluable patients (19 per group) after accounting for 20% attrition. While an effect size of 0.80 may be supported by prior pilot work (e.g., Ntoukas et al., 2023) and other exercise oncology literature, it remains optimistic. Can the authors elaborate on the rationale for selecting ES = 0.80 as the target? Would the authors consider including a sensitivity analysis to explore power implications for more modest effect sizes (e.g., ES = 0.50), which may be clinically relevant in this population? This would contextualize the feasibility of detecting smaller but still clinically meaningful effects in future trials.**

We believe that a large effect on strength is realistic in our study, given that we will compare HLST to no exercise. Additionally, study participants will not have participated in regular strength training activities for at least the past one-month. This is explained on page 21. In a future phase 3 trial comparing HLST to LMST, we would power for lower effects (i.e., ≤0.33) on strength and other patient-reported outcomes.

2. **The protocol notes an intention-to-treat analysis, but does not specify the approach for handling missing data due to attrition. How do the authors plan to handle missing outcome data if the attrition rate exceeds 20%? Will multiple imputation, mixed-effects modeling, or another method be applied to mitigate bias?**

Thank you for this comment. On Page 22 of the manuscript, we explain that if missing data is <10% we will conduct a complete case analysis. If missing data is >10%, we will employ a multiple imputation missing data strategy. There will be no interim analyses (i.e., at 6 weeks/midway through the 3-month intervention).

3. **The trial uses usual care (UC) as the control arm, which is methodologically acceptable for early-phase feasibility trials. Did the study team consider using light-to-moderate strength training (LMST) as a comparison group instead of UC? Given that LMST has previously shown benefits in this population, would this not serve as a more informative comparator for assessing the incremental benefit of HLST? A comparison against LMST might help determine whether HLST confers superior benefits, while also controlling for placebo and behavioral engagement effects.**

This is an important point. Yes, in the initial conception of this study, we considered comparing HLST to LMST. However, the preceding study included a small sample size of 9 participants. With this small dataset, we thought that it would be too big of a jump to go ahead and compare two interventions against each other. Instead, we have decided to confirm the feasibility of a HLST intervention in this larger study, while comparing it to no exercise, similar to a phase 2 study. A future phase 3 study would then compare HLST to the current standard of care (LMST) to determine the optimal exercise intensity for this population. We have now clarified this in the manuscript on page 9.

4. **This is a single-center trial conducted at the University of Alberta, which can streamline recruitment and intervention fidelity. However, it may limit the external validity and generalizability of the findings. Do the authors foresee challenges in generalizing results from a single site? Are there plans to scale this intervention to a multicenter context in future trials, and what barriers to implementation have been considered? A brief discussion of site-specific factors (e.g., infrastructure, clinical culture) that could influence reproducibility elsewhere would be useful.**

Although this study will be conducted at the University of Alberta, study participants will be recruited from all of Edmonton, AB, Canada, and surrounding areas. Exercise sessions take place in-person, so recruiting study participants to our study that live in other regions would not be realistic. However, we do agree that ideally, a multi-site centre trial would provide greater external validity and greater ability to generalize our findings to a wider head and neck cancer population.

In the future, we plan to scale this intervention to include multiple centres. If this current LIFTING 2 study establishes feasibility, safety, and efficacy, we can then estimate how many sites would be needed to properly power a phase 3 study. A multi-site study would then allow for a greater sample size, as well as greater validity and generalizability of our study findings. Potential barriers to implementation that may take place in a future multi-site study may include: variations in procedures and practices between sites, communication breakdown and challenges across different time zones, and standardization in exercise delivery methods may be challenging with various levels of experience and possible differences in protocol training conducted for exercise supervisors at each site. We now note this limitation and future plan in the manuscript on page 21 and 27.

5. **The trial uses stratified randomization based on sex and cancer stage (I–III vs. IV), a reasonable approach for ensuring balance in known prognostic categories. However, HNCS is a clinically heterogeneous group. Could the authors clarify whether any additional prognostic factors were considered during the design phase for inclusion in the randomization scheme or as covariates in the primary analysis? For instance, time since treatment completion, HPV status, baseline physical function, or dysphagia severity may influence strength or QoL outcomes.**

Given that the LIFTING 2 trial is a small phase 2 study, we could only consider 2 stratification factors. In the larger, phase 3 trial, we will consider additional stratification factors as you have pointed out, and have explained this in pages 8 and 9 of the manuscript.

During the pre-screening meeting and within the baseline questionnaires, HPV status (positive, negative, or unsure) and end of treatment date will be recorded within our database (REDCap).

Additionally, physical function and body composition measures such as upper and lower body maximal strength, grip strength, lean mass and body fat percentage will be assessed at baseline and postintervention (at the end of the 3-month intervention).

Lastly, patient-reported outcomes such as sleep quality, exercise behaviours, smoking and alcohol consumption status, swallowing difficulties, and levels of fatigue are assessed via validated and reliable questionnaires at baseline and postintervention. We do agree that various factors such as inadequate nutritional intake due to swallowing difficulties and relatively recent treatment completion dates may hinder one’s ability to increase strength, for example, compared to those with minimal swallowing difficulties and longer-term survivors. On page 22 of the manuscript, we have noted that covariates will be taken into account in the primary analysis.

6. **Due to the nature of the intervention, blinding of participants and investigators is not feasible. However, outcome assessor blinding (especially for strength testing) may still be possible and should be considered to reduce detection bias. Additionally, the use of objective measures alongside self-reported outcomes helps mitigate potential performance bias.**

This is an excellent suggestion and we have thought about this in depth. Due to logistical challenges at our centre, and the high likelihood that all members of our small lab will cross paths with exercising participants at some point (making them aware of their randomization), outcome assessor blinding is not feasible for us. However, we will be following a standardized protocol and the study coordinator will provide extensive training to other members who may be conducting exercise tests in her absence. We now note this limitation in the protocol on page 9.
